# Surgical reconstruction for congenital tracheal malformation and pulmonary artery sling

**DOI:** 10.1186/s13019-019-0858-2

**Published:** 2019-03-01

**Authors:** Huu Vinh Vu, Quang Khanh Huynh, Viet Dang Quang Nguyen

**Affiliations:** Department of Thoracic Surgery, Choray Hospital, Nguyen Chi Thanh street, District No. 5, Hochiminh City, Vietnam

**Keywords:** Congenital tracheal malformation, Slide tracheoplasty, Pulmonary artery sling

## Abstract

**Background:**

Congenital tracheal malformations are less common than congenital cardiac diseases and surgical repair of these anomalies is complex. We sought to examine the surgical treatment and outcomes in cases of tracheal anomalies presenting with or without associated congenital malformations.

**Methods:**

We retrospectively reviewed the demographic, clinical, and imaging data of 49 children who underwent surgery for congenital tracheal malformations between August 2013 and September 2017. Data were collected from the hospital records.

**Results:**

In all, 49 patients (male, 30; female, 19) underwent surgeries at our center. The children were of ages between 3 and 36 months (average: 9.7 months). Associated congenital lesions included sling in31/49 (63%), vascularring: in 2/49; ventriculoseptaldefectin5/49; Fallot’s tetraology in 2/49 (4.1%), and imperforate anus in 3/49 (6.1%). The outcomes of surgery were excellent in 42(85.7%) cases, good in 3 cases, while mortality occurred in 4(8.1%) cases. All cases of tracheal stenosis without any change in tracheobronchial arborization, 10/12 cases of bridge carina, and all cases of tripod carina were reconstructed using the slide tracheoplasty technique. Antetracheal translocation was performed for correction of associated pulmonary sling, without reimplantation of the pulmonary artery.

**Conclusions:**

Reconstructive surgery is a feasible treatment option for congenital tracheal malformations. Slide tracheoplasty can be safely applied in all cases for the correction of tracheal stenosis. Segment resection was not required for any portion of the trachea. Pulmonary artery translocation is safe and effective for patients with pulmonary artery sling, rather than reimplantation. Mortality was associated with severe cardiac complications.

## Background

Congenital tracheal stenosis is rare but it’s the main cause of airway obstruction in children, especially in infants. They are characterized by the presence of complete tracheal rings along the trachea with different lengths. Congenital tracheal stenosis is known to be associated with a high risk of severe complications that entail life-threatening episodes and may necessitate dependence on number of ventilator support and extracorporeal membrane oxygenation [1]. Hence, the diagnosis and management of congenital tracheal stenosis are importance.

Earlier, these lesions were classified on the basis of the clinical presentation associated with tracheal stenosis [2]. Subsequently, a newer classification system accounting for bronchial involvement was proposed on the basis of both the length of tracheal involvement as well as morphology. The four types were as follows: (1) normal arborization of the tracheobronchial tree, with the trachea bifurcating into the right and left bronchi at the carina; (2) a separate branch arising from the right bronchus to supply the right upper lobe; (3) three distinct bronchi arising from the carina; and (4) absence of one lung, associated with an absent or vestigial bronchus [3].

The actual incidence of congenital tracheal stenosis is not known and many cases end in fatality before the diagnosis is made [4]. Depending on the degree of tracheal stenosis, the clinical manifestations of tracheal malformations may range from stridor episodes to severe respiratory failure; asymptomatic patients have also been reported [5, 6]. Generally, echocardiography and three-dimensional reconstruction of computed tomography images are necessary to evaluate patients with congenital tracheal stenosis for the identification of the associated cardiovascular anomalies [6]. Congenital tracheal stenosis usually occurs in conjunction with other congenital cardiac and/or vascular anomalies, such as pulmonary artery sling and vascular rings. The management of the various combinations of these congenital anomalies is challenging. Although some there has been some evidence of the spontaneous resolution of the respiratory distress associated with some tracheal abnormalities in children, the general consensus is to advise surgery if the child develops severe distress before the completion of one year [6]. Surgical reconstruction is the treatment of choice for severe cases of airway obstruction; however, the rate of mortality continues to remain significant. Therefore, the optimal treatment still remains debatable.

In this study, we sought to evaluate the safety and effectiveness of surgical correction of tracheal stenosis and associated vascular abnormalities by means of a single-center study conducted over period of five years.

## Methods

### Study design and subjects

The study was planned as a retrospective investigation of the data of all patients who underwent surgical repair of congenital tracheal malformations performed by the same surgeon at our center, between August 2013 and July 2017. The study protocol was approved by the institutional review board. Since this was a retrospective surgery, the need for consent was waived.

### Data collection

The patients’ data were collected from the hospital records. Data were collected for the following parameters: age, sex, clinical presentation, type of tracheobronchial malformation, associated congenital abnormalities, type of surgery applied, number of surgeries required, and postoperative outcomes. The first and most important parameters are the patient’s survival and life. Most of the cases that failed in surgery would be paid by patient’s life. They can’t survive without repair surgery or unsuccessful repair operation. The survival patients were required to have repeated examinations periodically, in order to evaluate the outcome of surgery and/or whenever they have symptoms requiring hospitalization, until they are free of symptom/hospitalization and completely return to their normal life. Ct-scan (even 3D reconstruction of the bronchus) and bronchoscopy are the most commonly used tests in several initial examinations. The internal caliber and shape of the trachea and main bronchus and the degree of dyspnea are the most parameter to concern. The pre- and postoperative images acquired by 3D reconstruction of CT scans were used for comparison of the pre- and postoperative tracheobronchial architecture. The data were expressed as percentages. Later, most of them were found to be free of symptom and did not need to have strict observation and examination.

## Results

### Demographic characteristics

In all, 49 infants underwent surgical repair of congenital tracheal anomalies performed by the same surgeon at our center during the study period. The ages of the infants ranged from 3 to 34 months (average age: 9.7 months) and 30 (61.2%) of the infants were male.

### Morphology

**Fig. 1:**
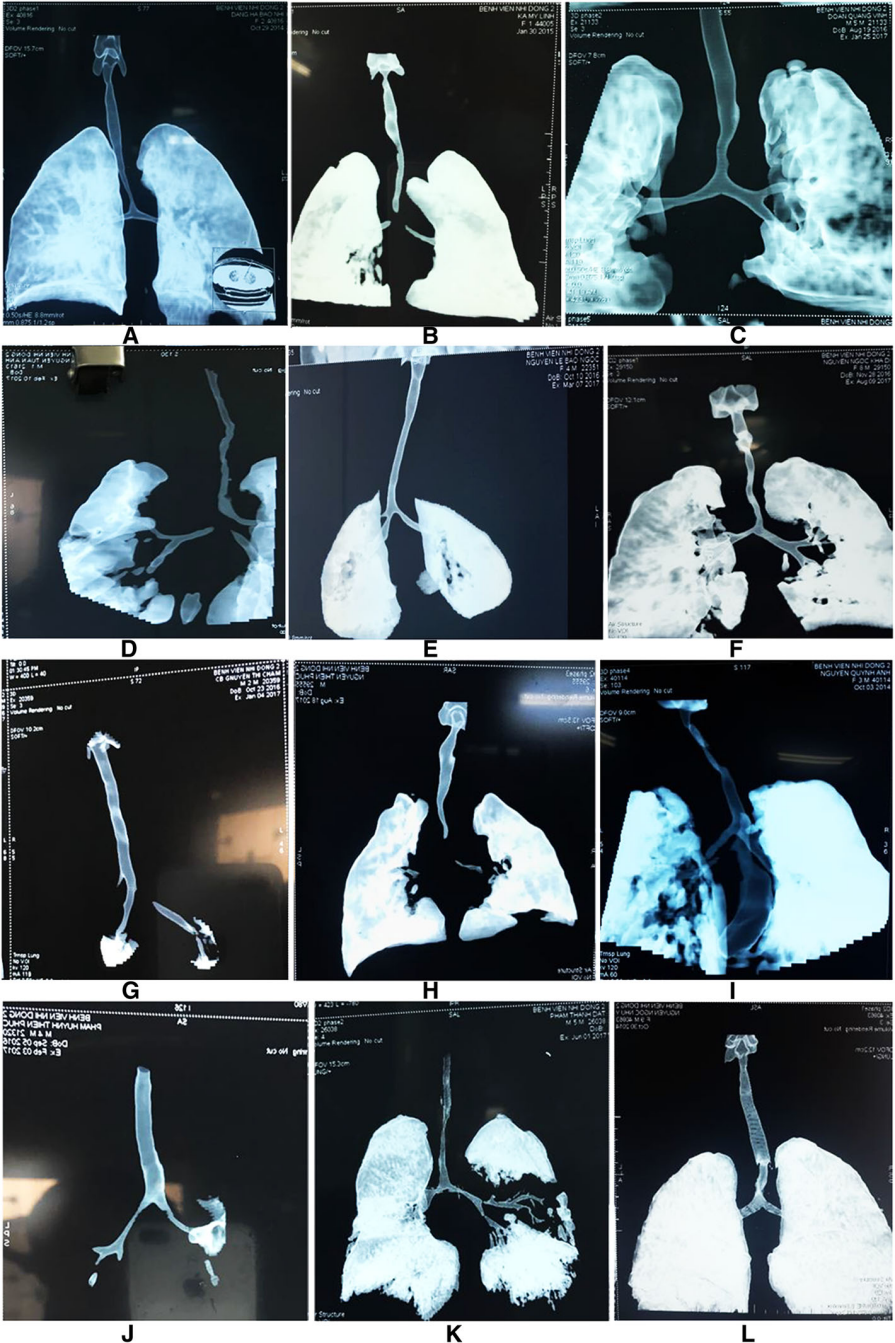
Congenital malformation of the trachea is very diversity in morphology. **a** Stenosis just above the carina; **b** Stenosis at the carina; **c** Stenosis at trachea and Rt. main bronchus; **d** Stenosis all length of trachea including carina; **e** Stenosis all length of trachea with tripod carina; **f** Stenosis 2/3 lower part of trachea and Rt. main bronchus; **g** Double carina with stenosis at tracheal bronchus and Lf main bronchus; **h** Stenosis at carina and both main bronchi; **i** Stenosis at 1/2 upper part of trachea; **j** Stenosis at both main bronchi; **k** Stenosis all trachea with severe stenosis point at the middle; **l** Sudden stenosis just above the carina

**Fig. 2:**
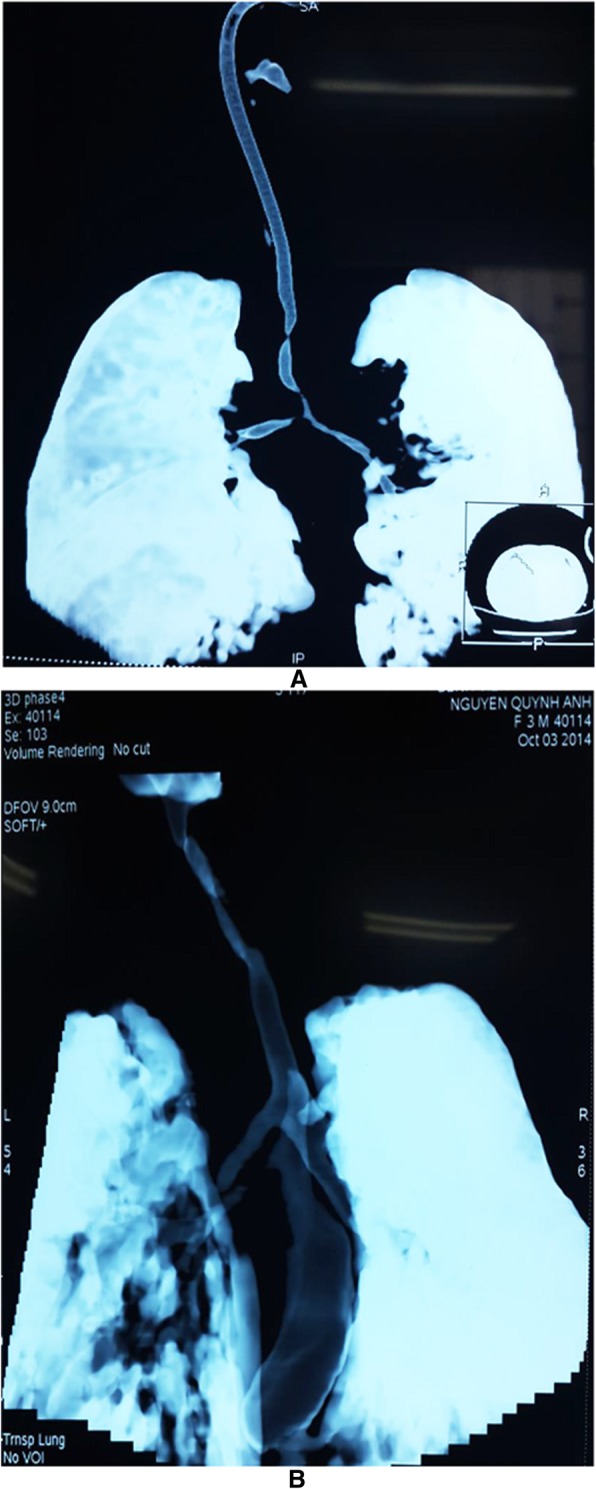
Normal shape but stenosis at some part of trachea, **a** Sausage like stenosis trachea; **b** Stenosis at 1/2 upper part of the trachea

**Fig. 3:**
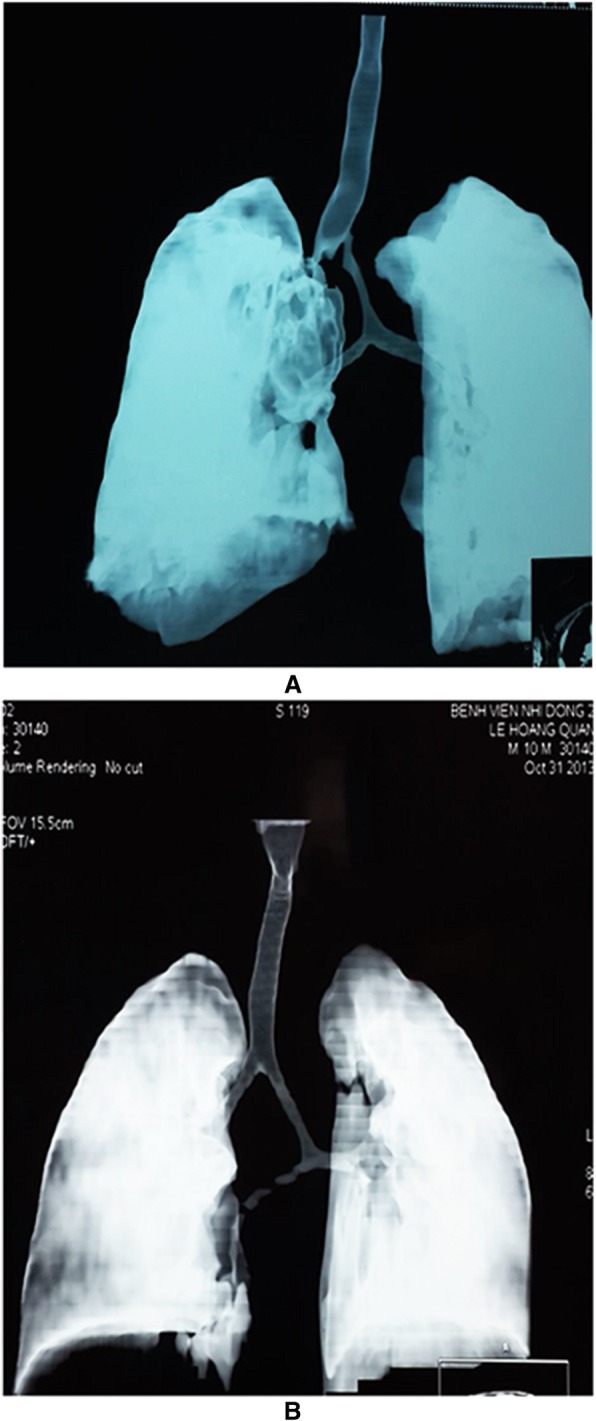
Double carina or bridge bronchus, **a** Tracheal bronchus is stenosis; **b** Rt. main bronchus is stenosis

**Fig. 4:**
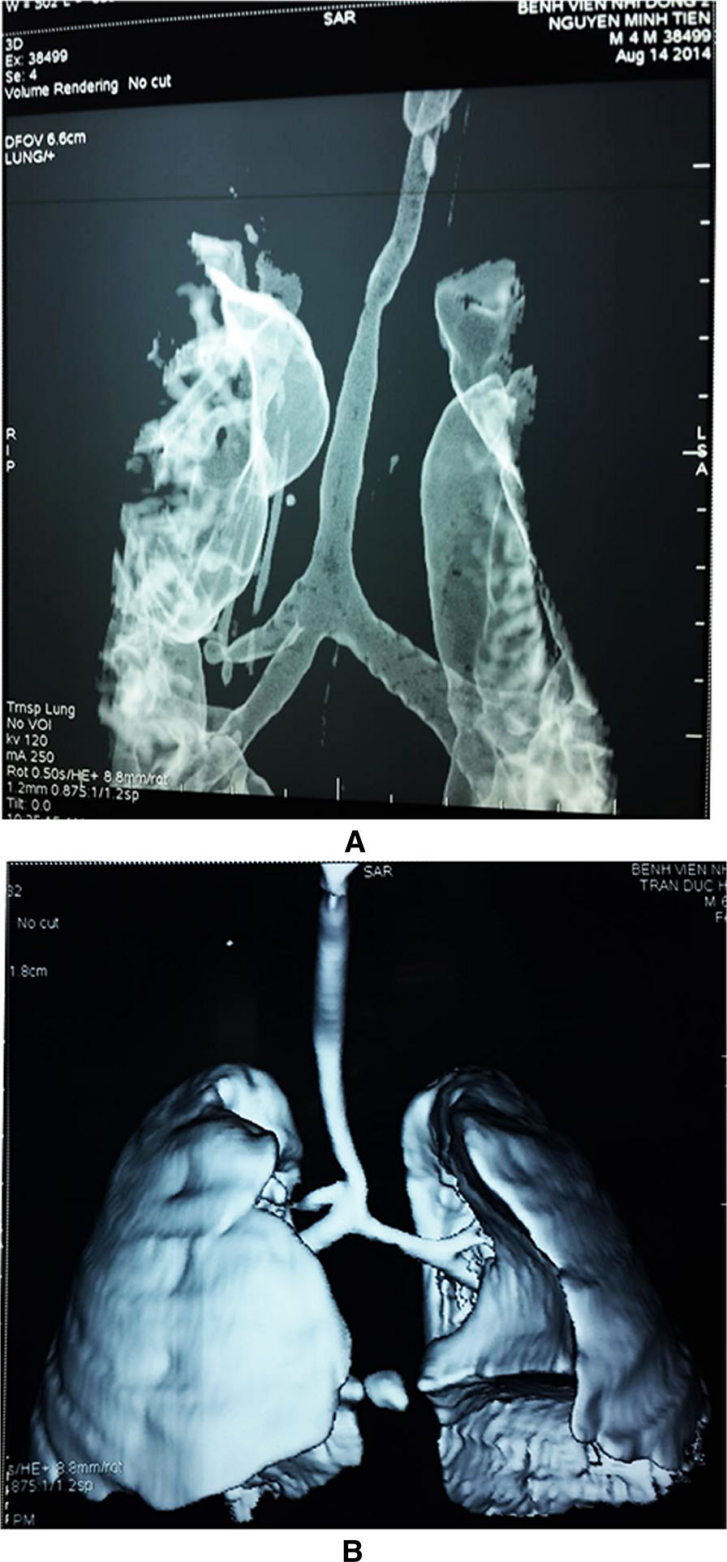
Tripod bronchus, three main bronchus arrive at the same level from trachea like a tripod, **a** Tripod bronchus with stenosis at upper part of trachea; **b** Tripod bronchus with stenosis at lower part of trachea

We observed various types of congenital tracheal malformations in terms of trachea bronchial morphology (Fig. 1a-l). Stenosis occurring in some tracheal regions without any change in the shape of the trachea was observed in 31 cases (Fig. 2a and b). Tracheal stenosis with double carina or bridge bronchus, wherein the right upper lobe has its own bronchus arise directly from the trachea (also termed tracheal bronchus) and is the most proximal one. Below that, or more distantly, there are two main bronchi, one to the left lung and one to the right middle and lower lobes (Fig. 3a and b). We termed it double carina because it looks like the trachea has two carinas connecting to each other by a bridge termed bridge bronchus. This type was observed in 12 cases. Further, tracheal stenosis with tripod carina, wherein the tracheal bronchus to the upper lobe of the right lung originated at the same level as the left bronchus and the bronchus to the middle lobe and lower lobe of the right lung, was observed in 6 cases. The origination of the three bronchi from the same tracheal level caused the trachea to appear similar to a tripod (Figs. 4a and b).

### Vascular anomalies

**Fig. 5:**
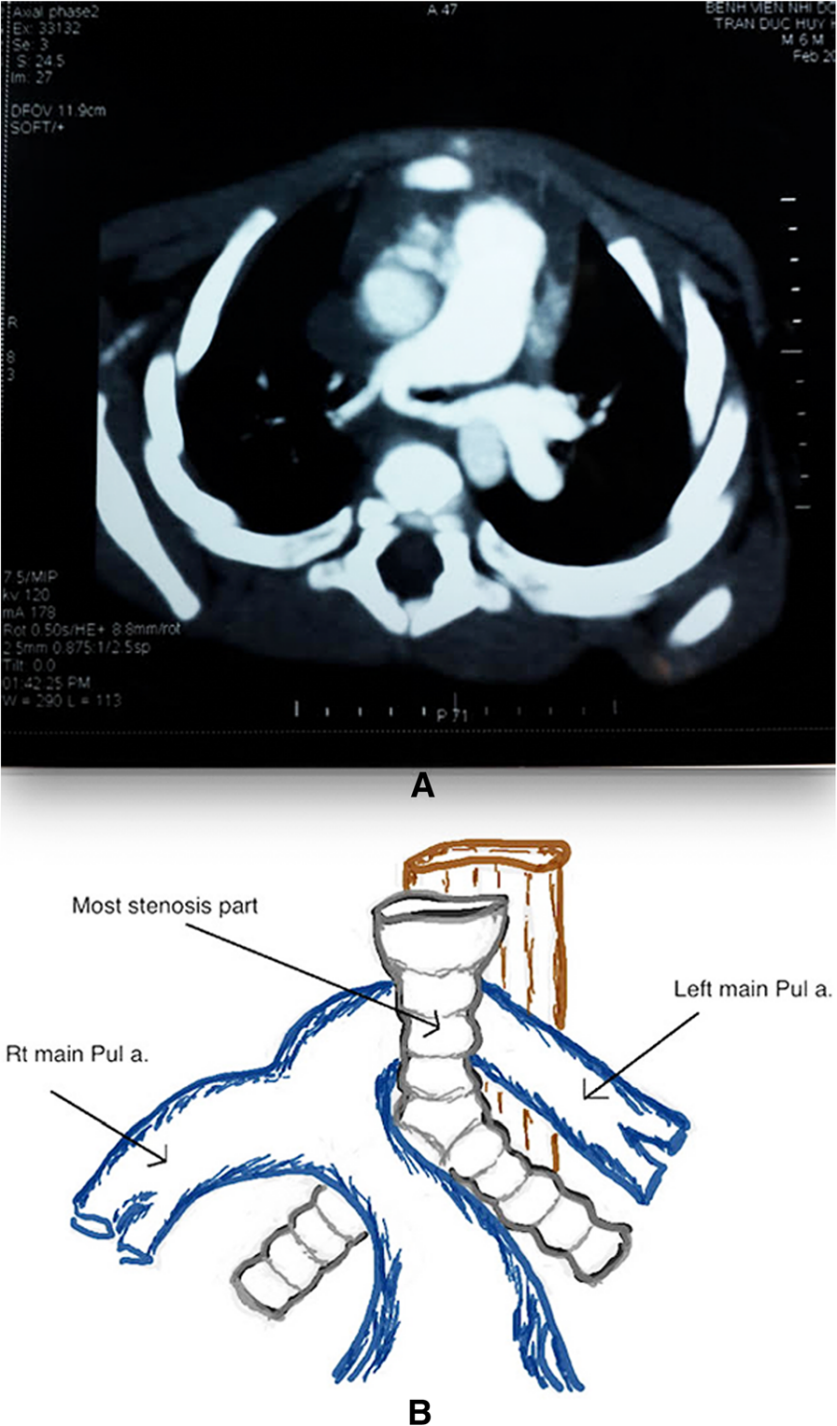
(**a** and **b**) CT scan show left main Pulmonary artery sling arrive from the right main Pulmonary artery sling

In addition to tracheal stenosis, 41 patients had other associated congenital defects. Pulmonary sling was the most common associated anomaly, occurring in 31 patients (Figs. 5a and b). Besides pulmonary artery sling, other associated congenital anomalies noted were vascular ring in 2 children, ventriculoseptal defect in 5, and imperforate anus in 3.

### Surgical treatment and outcome

All patients with stenosis but normal trachea (type 1) and tripod carina (type 3) underwent repair surgery using sliding technique, while 10 (83.3%) of the 12 cases of bridge bronchus (double carina, type 2) were treated with this sliding technique. The remaining two cases of bridge bronchus required resection and reanastmosis. For all patients with associated pulmonary artery sling, antetracheal translocation of the pulmonary artery was performed, without reimplantation. All patients underwent corrective surgery under ventilatory support provided with the heart-lung machine. In 47 of the cases, only one surgery was performed, while the remaining two required repeat surgery due to restenosis. Of the 47 patients who underwent a single surgical procedure, 44 survived and 43 recovered well with achievement of good caliber and normal shape of the trachea as well as restoration of the pulmonary trunk and shape and function of the pulmonary artery. One had good recovery with restoration of the normal caliber and shape of the trachea, but continued to have mild episodes of stridor after the operation. The remained three patients died in the post-operative care unit due to severity of the respiratory distress and cardiac failure. Two patients underwent repeated surgeries. Both of them were on ventilator with severe respiratory distress before surgery. Both patients were on ventilator support. Those two patients had stenosis but not so severely followed by critical stages due to postponing and delay in surgical intervention decision. Also, we didn’t get any good result after the first surgery and were considered for redo (second) surgery. Both of the patients had severe respiratory distress and even cardiac arrest occurring before surgery and on ventilation support. The severity of the patient compromised surgeon manipulation during the first operation. One died after redo surgery, one survived with good result.

### Mortality

Three patients died after the first surgery due to severe stenosis after repair surgery. We didn’t get any chance for them to have second (redo) surgery. There were no intraoperative deaths among our sample population. Two patients died due to late events that occurred in the intensive care unit. One of the critically ill patients who required repeat surgery died after the second surgery.

## Discussion

This retrospective study on congenital tracheal stenosis conducted over a period of five years revealed the following: there were three major types of morphological presentations of the tracheobronchial tree; slide tracheoplasty was a safe, effective, and versatile procedure suitable for application in various types of clinical presentations; and all cases with pulmonary artery sling could be managed by translocation, precluding the need for reimplantation and reanastomosis; mortality was associated with long stenosis part, other cardiac anomalies and the need for repeat surgery and severe respiratory distress necessitating ventilator support.

In our study, we found three types of tracheobronchial morphology: (1) stenosis in some parts of the trachea, without any change in the shape of the trachea; (2) stenosis due to double carina, bridge bronchus; (3) stenosis with tripod carina. These are consistent with the first three types of the classification system proposed by Speggiorin et al. (2011) [7]. There were no cases of the fourth type, i.e., absent lung among our patients.

The choice of the correct surgical procedure is made on the basis of the type of malformation, although slide tracheoplasty is a versatile procedure [8]. We successfully applied the slide tracheoplasty technique and obtained excellent outcomes in all cases of tripod carina, almost all cases of bridge carina (10/12), and all cases of tracheal stenosis without any change in tracheal shape. This finding is consistent with those of previous studies that have shown the feasibility of slide tracheoplasty in the management of congenital tracheal stenosis [3, 9–11]. Mortality and morbidity associated with this technique has been reported to be comparatively low, as observed in our study [11].

Further, repeat surgery was required in 2 of the 49 cases, which corresponds to 4%. This percent is less than that reported in a similar study, where the percentage of repeat surgery due to restenosis was 11% [9]. Both these patients were clinically in critical situations, with multiple cardiac arrests occurring before surgery and both requiring ventilator support. One (50%) of these patients died after surgery, which implies that critical clinical status necessitating ventilator support would be a risk factor for poor prognosis. However, further investigation would be necessary to confirm the relationship between ventilator support, reoperation, and poor prognosis in the surgical treatment of congenital tracheal stenosis.

In all our patients with associated pulmonary artery sling, we used the antetracheal translocation technique and obtained excellent results. Some previous studies suggest the superiority of reimplantation technique over antetracheal translocation [12]. However, that study was based only on two cases and ours included 31 cases in which the results of translocation were highly successful. Moreover, with time, there have been improvements in the perioperative investigations, which may contribute towards the better results noted in our study. To our knowledge, it appears that this study presents the most large-scale evidence in support of antetracheal translocation in cases of tracheal stenosis with pulmonary artery sling. For pulmonary artery sling, some investigators recommend that left pulmonary artery reimplantation with slide tracheoplasty is beneficial [13]. However, this study was highly criticized [14]. Our experience with antetracheal translocation of the pulmonary artery over the period of five years and in 31 cases of tracheal stenosis with pulmonary artery sling provides strong evidence in favor of the procedure. We recommend that the choice of this procedure be made under careful condition and by correlating with the patient’s clinical status.

Our study revealed that surgical reconstruction of congenital tracheal stenosis is safe and effective, especially before the onset of severe respiratory distress necessitating the initiation of ventilator support. Four of our cases ended in fatality: three of these died from events that occurred in the postoperative intensive care unit, whereas one required a second operation, after which the child died.

With respect to the clinical status of the patients, the general consensus is that aggressive surgical intervention is necessary in patients, who develop severe respiratory distress within the first year of life [6]. Our patients present with a range of clinical manifestations, ranging from mild stridor to severe respiratory distress. Poor outcomes were observed in patients who already had cardiac complications such as cardiac arrests and required ventilator support.

One of the merits of this study is that since all the surgeries in this study were performed by the same surgeon, we were able to eliminate the effects of inter-operator variability on the outcome of surgery. This adds further value to our results.

Some of the limitations of this study are the small sample size and retrospective design. However, this is one of the first studies on surgical treatment of tracheal malformations from our region. Moreover, very few studies have been available on the effectiveness of antetracheal translocation. In future, large-scale studies are necessary in future to gain further insights into the causes of the malformations and the associations with.

## Conclusion

From our experiences in this study, surgical repair of tracheal malformations appears to yield favorable results in young children. From the above study we concluded that, slide tracheoplasty in addition to the vascular/cardiac supportive procedures are effective for similar patients and is associated with good outcomes. We also found that antetracheal translocation; along with slide tracheoplasty was infective in management of pulmonary sling. Further study to confirm the effectiveness of this procedure on a more large-scale population and for a longer duration is warranted.

## References

[1] Antón-Pacheco JL, Cano I, García A, Martínez A, Cuadros J, Berchi FJ (2003). Patterns of management of congenital tracheal stenosis. J Pediatr Surg.

[2] Antón-Pacheco JL, Kaliciński P, Kansy A, Maruszewski P (2012). Slide tracheoplasty in an infant with congenital tracheal stenosis and oesophageal atresia with tracheoesophageal fistula. Eur J Cardiothorac Surg.

[3] ButlerCR SS, Rijnberg FM (2014). Outcomes of slide tracheoplasty in 101 children: a 17-year single-center experience. JThoracCardiovasc Surg.

[4] Chung SR, Yang JH, Jun TG (2015). Clinical outcomes of slide tracheoplasty in congenital tracheal stenosis. Eur J Cardiothorac Surg.

[5] Elliott M, Roebuck D, Noctor C (2003). The management of congenital tracheal stenosis. Int J PediatrOtorhinolaryngol.

[6] Herrera P, Caldarone C, Forte V (2007). the current state of congenital tracheal stenosis. PediatrSurg Int.

[7] Hofferberth SC, Watters K, Rahbar R, Fynn-Thompson F (2015). Management of Congenital Tracheal Stenosis. Pediatrics.

[8] Hong X, Zhou G, Liu Y, Liu Y, Wang H, Feng Z (2015). Management of pulmonary artery sling with tracheal stenosis: LPA re-implantation without tracheoplasty. Int J ClinExp Med.

[9] Licari A, Manca E, Rispoli GA, Mannarino S, Pelizzo G, Marseglia GL (2015). Congenital vascular rings: a clinical challenge for the pediatrician. PediatrPulmonol..

[10] Manning PB, Rutter MJ, Lisec A, Gupta R, Marino BS (2011). One slide fits all: the versatility of slide tracheoplasty with cardiopulmonary bypass support for airway reconstruction in children. J ThoracCardiovasc Surg.

[11] Speggiorin S, Torre M, Roebuck DJ, McLaren EMJ (2011). Surgical outcome of slide tracheoplasty in patients with long congenital segment tracheal stenosis and single lung. EurJCardiothorac Surg.

[12] Speggiorin S, Torre M, Roebuck DJ, McLaren CA, Elliott MJ (2012). A new morphologic classification of congenital tracheobronchial stenosis. AnnThorac Surg.

[13] van Son JA, Hambsch J, Haas GS, Schneider P, Mohr FW (1999). Pulmonary artery sling: reimplantation versus antetracheal translocation. Ann Thorac Surg.

[14] Torre M (2017). Left pulmonary artery sling and congenital tracheal stenosis: to slide or not to slide?. J Thorac Dis.

